# Low Zika Virus Seroprevalence in Vientiane, Laos, 2003–2015

**DOI:** 10.4269/ajtmh.18-0439

**Published:** 2019-01-28

**Authors:** Boris Pastorino, Onanong Sengvilaipaseuth, Anisone Chanthongthip, Manivanh Vongsouvath, Chanthala Souksakhone, Mayfong Mayxay, Laurence Thirion, Paul N. Newton, Xavier de Lamballerie, Audrey Dubot-Pérès

**Affiliations:** 1 Unité des Virus Émergents (UVE: Aix-Marseille Univ–IRD 190–Inserm 1207–IHU Méditerranée Infection), Marseille, France;; 2 Lao-Oxford-Mahosot Hospital-Wellcome Trust Research Unit (LOMWRU), Microbiology Laboratory, Mahosot Hospital, Vientiane, Lao PDR;; 3 National Blood Transfusion Centre, Lao Red Cross, Vientiane, Lao PDR;; 4 Institute of Research and Education Development, University of Health Sciences, Vientiane, Lao PDR;; 5 Centre for Tropical Medicine and Global Health, Nuffield Department of Medicine, Churchill Hospital, University of Oxford, Oxford, United Kingdom

## Abstract

Zika virus (ZIKV) has been presumed to be endemic in Southeast Asia (SEA), with a low rate of human infections. Although the first ZIKV evidence was obtained in the 1950s through serosurveys, the first laboratory-confirmed case was only detected in 2010 in Cambodia. The epidemiology of ZIKV in SEA remains uncertain because of the scarcity of available data. From 2016, subsequent to the large outbreaks in the Pacific and Latin America, several Asian countries started reporting increasing numbers of confirmed ZIKV patients, but no global epidemiological assessment is available to date. Here, with the aim of providing information on ZIKV circulation and population immunity, we conducted a seroprevalence study among blood donors in Vientiane, Laos. Sera from 359 asymptomatic consenting adult donors in 2003–2004 and 687 in 2015 were screened for anti-ZIKV IgG using NS1 ELISA assay (Euroimmun, Luebeck, Germany). Positive and equivocal samples were confirmed for anti-ZIKV–neutralizing antibodies by virus neutralization tests. Our findings suggest that ZIKV has been circulating in Vientiane over at least the last decade. Zika virus seroprevalence observed in the studied blood donors was low, 4.5% in 2003–2004 with an increase in 2015 to 9.9% (*P* = 0.002), possibly reflecting the increase of ZIKV incident cases reported over this period. We did not observe any significant difference in seroprevalence according to gender. With a low herd immunity in the Vientiane population, ZIKV represents a risk for future large-scale outbreaks. Implementation of a nationwide ZIKV surveillance network and epidemiological studies throughout the country is needed.

## INTRODUCTION

Zika virus (ZIKV) is a *Flavivirus* that was first isolated in Uganda from a sentinel monkey in 1947, and then 1 year later from *Aedes* mosquitoes.^1^ The first ZIKV isolation from a patient occurred in 1952 in Nigeria.^2^ Serological evidence suggests that ZIKV spread throughout Africa and Asia but that its circulation went largely undetected.^3^ Indeed, only 16 human cases were confirmed before the first known outbreak in 2007 in Yap island.^4^ A second outbreak occurred in French Polynesia in 2013–2014, rapidly spreading to other Pacific Islands where it was still circulating in 2015.^5^ Probably introduced from Pacific Islands in the second half of 2013,^6,7^ ZIKV transmission was confirmed in Brazil in May 2015 and caused a major outbreak, which extended in 2016 to other countries in South and Central America, and the Caribbean.^8^

In Southeast Asia (SEA), ZIKV was first isolated from mosquitoes in Malaysia in 1966.^9^ First evidence of ZIKV circulation in human was obtained from serosurveys, using neutralization assays, in the early 1950s (Philippine 1953, Malaysia 1953–1954, Thailand 1954, and Vietnam 1954).^3^ However, the first human laboratory-confirmed patient was only recently reported, in 2010 from Cambodia.^10^ Human ZIKV infections have probably been underreported because of confusion with other febrile illness and difficulty in accessing laboratory assays.^11^ Recent large ZIKV outbreaks in the Pacific Islands and the Americas encouraged investigations in SEA. In the past few years, mainly from 2016, many Asian countries began to report ZIKV-infected patients demonstrating a wide geographical spread of the ZIKV Asian lineage.^12^ In Thailand, nine cases were reported between 2012 and 2014, followed by 686 in 2016. In Vietnam, two cases were reported in 2013, followed by 219 in 2016, and 13 in the first 2 months of 2017. In Singapore, the first autochthonous case was confirmed in August 2016; 493 cases were then reported as of June 2017. In Philippines, 58 cases have been reported from 2012. In Indonesia, two cases were reported in 2015. In Cambodia, after the first report in 2010, a retrospective study confirmed five cases between 2007 and 2015 and one case was reported in November 2016. In Malaysia, two cases were reported in 2016. In Myanmar, ZIKV was confirmed in one expatriate in 2016. In China, only imported cases have been reported. In Lao PDR, 18 confirmed patients were detected from a retrospective analysis of sera collected in 2012–2013 and one patient was reported from 2016.^12^

It is difficult to determine if the recent increase in reported cases is due to improved surveillance or to an actual increase in human cases in the region. Zika virus has been presumed to be endemic with a low rate of human infections in SEA, but its actual epidemiological status and the ecological determinants of its long-term maintenance remain uncertain because of the scarcity of available information.^13^ Zika virus circulation may also be underestimated because of the asymptomatic nature of a large proportion of human infections combined with clinical presentations sharing similarities with other endemic febrile diseases.^12^ In consequence, the level of herd immunity in Asian populations and, therefore, the risk of epidemic spread of ZIKV are unknown.

Zika virus circulation in Vientiane in the past decade has probably been underestimated because of the lack of specific surveillance. Here, we report a seroprevalence study in Vientiane capital in asymptomatic blood donors sampled in 2003–2004 and 2015 to document ZIKV circulation and population immunity level.

## MATERIAL AND METHODS

### Samples.

Two milliliter of whole blood were collected by the National Blood Transfusion Centre of the Lao Red Cross from asymptomatic adult blood donors, 359 samples from 2003 to 2004, and 687 samples from 2015. All donors gave written informed consent to be included in this study. Ethical approval was granted by Lao National Ethics Committee for Health Research and the Oxford Tropical Research Ethics Committee. After blood centrifugation, sera were aliquoted and stored at −80°C for testing.

### Laboratory assays.

All sera were tested by the anti-ZIKV NS1 IgG ELISA kit (Euroimmun, Luebeck, Germany) following manufacturer’s instruction. Positive and equivocal samples were then tested by a virus neutralization test (VNT) performed in a 96-well format, using Vero cells (ATCC number CCL-81) and 50 pfu of ZIKV (strain MRS-OPY_Martinique_Pari_2015 belonging to Asian lineage), as described.^14^ A threshold titer of 40 was used for positivity, as recommended by the French National Reference Centre for Arboviruses. The same strategy (ELISA + VNT) has been used in previous ZIKV seroprevalence studies in Cameroon,^15^ Martinique Island,^14^ and Bolivia.^16^ The study in Martinique island, where dengue seroprevalence is greater than 90%, involved testing of blood donors before and after the 2016 Zika outbreak. This provided evidence that the strategy of ELISA + VNT yields high specificity and sensitivity, greater than 98% with reference to 90% end point plaque reduction neutralization test, even in dengue-endemic contexts.^17^

### Statistical analyses.

Analyses were conducted using STATA/SE, v14.0 (StataCorp, College Station, TX).

## RESULTS

Zika virus seroprevalence was defined as the proportion of donors with positive ZIKV neutralization tests. Anti-ZIKV IgG ELISA assays were performed on all sera. Positive and equivocal sera by ELISA (62 [17.3%] and 20 [5.6%], respectively, in 2003–2004, 192 [27.9%] and 27 [3.9%], respectively, in 2015) were then submitted for VNT.

Among 359 sera from 2003 to 2004, neutralizing anti-ZIKV antibodies were confirmed in 16 donors (4.5%, 95% CI: 2.6–7.1) (Table 1

**Table 1:** Characteristics of blood donors and ZIKV seroprevalence for the two study periods, 2003–2004 and 2015

	2003–2004 (*n* = 359)		2015 (*n* = 687)	
Gender ratio, male/female	280/72 (3.9)		425/256 (1.7)	
Age (years), median	20		28	
Interquartile range	18–23		23–35	
Range	16–63		17–79	
Laboratory assays				
Anti-Zika IgG ELISA result*, *n* (%)	Positive	Equivocal	Positive	Equivocal
	62/359 (17.3%)	20/359 (5.6%)	192/687 (27.9%)	27/687 (3.9%)
Zika VNT positive, *n* (%)	13/62 (21.0%)	3/20 (15.0%)	62/192 (32.3%)	6/27 (22.2%)
ZIKV seroprevalence				
Number of positive/total number	16/359		68/687	
Percentage (95% CI)	4.5% (2.6–7.1)		9.9% (7.8–12.4)	
ZIKV seroprevalence in females				
Number of positive/total number	3/72		25/256	
Percentage (95% CI)	4.2% (0.9–11.7)		9.8% (6.4–14.1)	
ZIKV seroprevalence in males				
Number of positive/total number	11/280		43/425	
Percentage (95% CI)	3.9% (2.0–6.9)		10.1% (7.4–13.4)	

IQR = interquartile range; ZIKV = Zika virus; VNT = virus neutralization test. ZIKV seroprevalence = proportion of donors with positive ZIKV neutralization tests; CI = confidence interval.

**Figure 1.**
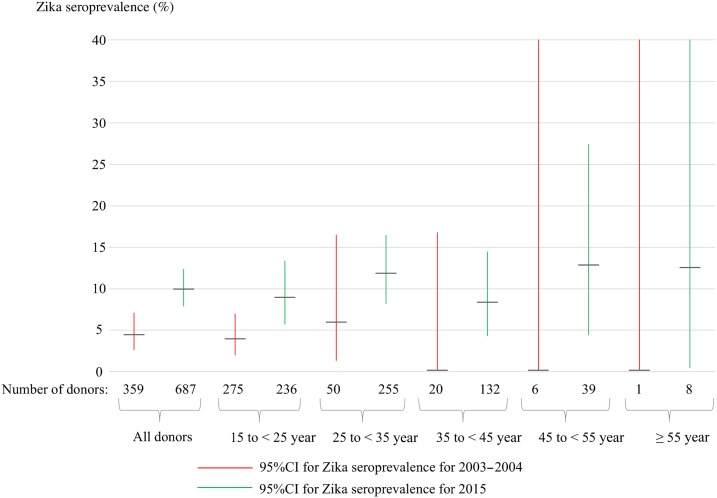
Comparison of Zika seroprevalence between the two study periods (2003–2004 and 2015), by 10-year age brackets. Zika seroprevalences and 95% CI, displayed as black bars for 2003–2004 and grey bars for 2015, are presented for all donors and for donors stratified in five age groups (15 to less than 25 years old, 25 to less than 35 years old, 35 to less than 45 years old, 45 to less than 55 years old, and equal or more than 55 years old). This figure appears in color at www.ajtmh.org.

* Result of anti-Zika IgG ELISA (Euroimmun) test following manufacturer’s instruction.

). A significant increase (*P* = 0.002, *z*-test) in ZIKV seroprevalence was observed in the 2015 population, with neutralizing anti-ZIKV antibodies confirmed in 68 donors (9.9%: 7.8–12.4). Blood donors were significantly older (*P* < 0.001, Mann–Whitney *U*-test) in 2015 than in 2003–2004 (Table 1, Figure 1, Supplemental Figure 1). However, significant increase in ZIKV seroprevalence was also observed in donors aged < 25 years old (Figure 1), from 4.0% (95% CI: 2.0–7.0) in 2003–2004 to 8.9% (95% CI: 5.6–13.3) in 2015 (*P* = 0.023).

Sex ratios (male:female) of the studied populations were 3.9 in 2003–2004 and 1.7 in 2015 (Table 1). No significant difference in ZIKV seroprevalence was observed between males and females during the two studied periods (Table 1, *P* = 0.927 in 2003–2004, *P* = 0.882 in 2015, chi square test).

## DISCUSSION

Our study provides evidence of ZIKV circulation in Vientiane capital, Laos, over the last decade, in line with the previous identification of 18 confirmed ZIKV viremic patients in 2012–2013 in the capital.^12^ The observed ZIKV seroprevalence in adult blood donors in Vientiane capital appeared to increase over time. Although precise epidemiological analysis was hampered by the fact that age distributions were not similar in our two successive serological studies, ZIKV seroprevalence appeared to increase for all age groups between 2003 and 2004 and 2015 (Figure 1, Supplemental Figure 1), most probably indicating an increase in ZIKV incidence in the population. The overall seroprevalence frequency observed remains low (< 10% seropositivity), suggesting limited ZIKV circulation. Accordingly, in the relatively immunologically naive Lao population and in the presence of a favorable climate for the rapid breeding of the vectors,^18^ ZIKV represents a real public health threat with a risk for future large-scale outbreaks. In this context, the potential role of the high levels of immunity within the SEA populations against other flaviviruses (dengue virus and Japanese encephalitis virus) is unknown.

In this study, no questionnaire was administrated to the blood donors consenting to participate. In the absence of detailed epidemiological, environmental, and behavioral information, these data do not enlighten us as to the actual situation of ZIKV transmission in Laos. In Vientiane capital, it is likely that ZIKV circulation follows an (*Aedes* mosquito–human–*Aedes* mosquito) urban transmission cycle. However, in more remote rural areas of the country, the virus may be maintained in sylvatic or (peri-)sylvatic enzootic cycles of transmission which remain entirely uncharacterized.

Here, we have provided the first recent ZIKV seroprevalence data in a SEA country. This study was performed using samples from the mostly urban area of Vientiane capital. The situation is likely to be different in rural areas and between the north and the south of the country, presenting significant topographic and climate variability. Therefore, extensive epidemiological studies in those different contexts are required to identify factors affecting ZIKV transmission and the role played by the co-circulation of other flaviviruses.

We conclude that it is of great importance to reinforce epidemiological and environmental studies of ZIKV in Southeast Asia to allow a better understanding of the maintenance and transmission cycles of the virus, to help mitigate the risk of future outbreaks and improve the medical management of at-risk populations.

## Supplementary Files

Supplemental figure
