# Supplementary material for: Low Zika Virus Seroprevalence in Vientiane, Laos, 2003–2015
Source: Am J Trop Med Hyg. 2019 Jan 28;100(3):639–42. doi: 10.4269/ajtmh.18-0439 (PMC6402904; doi:10.4269/ajtmh.18-0439)

The following are supplemental materials and will be published online only

S1 Figure: Comparison of Zika seroprevalence between the 2 studied periods (2003-2004 and 2015), by 5-year age brackets. Zika seroprevalences and 95%CI, displayed as red bars for 2003-2004 and green bars for 2015, are presented for all donors and for donors stratified in 8 age groups (15 to less than 20 year old, 20 to less than 25 year old, 25 to less than 30 year old, 30 to less than 35 year old, 35 to less than 40 year old, 40 to less than 45 year old, 45 to less than 50 year old and equal or more than 50 year old).

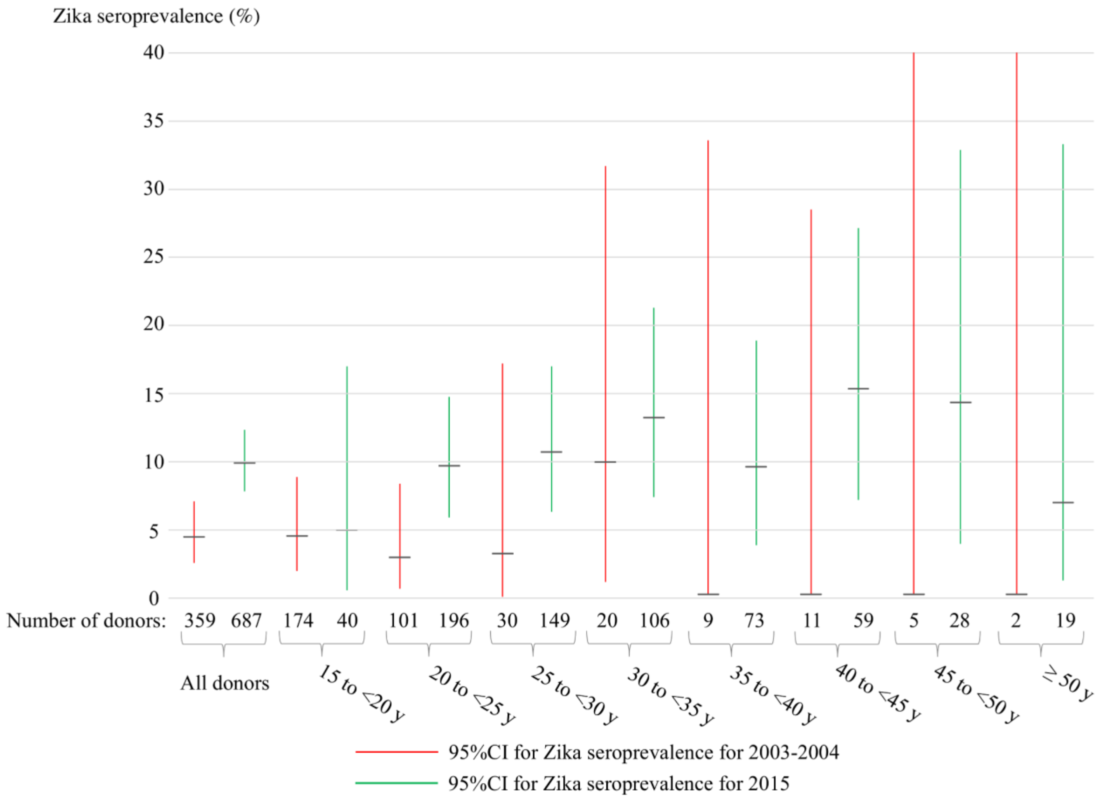

Supplement: Supplementary file 1 [file tpmd180439.SD1.pdf]
